# Anisotropy and Strain Localization in Dynamic Impact Experiments of Tantalum Single Crystals

**DOI:** 10.1038/s41598-018-23879-1

**Published:** 2018-04-03

**Authors:** Hojun Lim, Jay D. Carroll, Corbett C. Battaile, Shuh Rong Chen, Alexander P. Moore, J. Matthew D. Lane

**Affiliations:** 1 Department of Computational Materials and Data Science, Sandia National Laboratories, Albuquerque, New Mexico 87175 USA; 2 Department of Materials Mechanics and Tribology, Sandia National Laboratories, Albuquerque, New Mexico 87175 USA; 3 Los Alamos National Laboratory, Los Alamos, New Mexico 87545 USA

## Abstract

Deformation mechanisms in bcc metals, especially in dynamic regimes, show unusual complexity, which complicates their use in high-reliability applications. Here, we employ novel, high-velocity cylinder impact experiments to explore plastic anisotropy in single crystal specimens under high-rate loading. The bcc tantalum single crystals exhibit unusually high deformation localization and strong plastic anisotropy when compared to polycrystalline samples. Several impact orientations - [100], [110], [111] and [$\bar{1}49$] - are characterized over a range of impact velocities to examine orientation-dependent mechanical behavior versus strain rate. Moreover, the anisotropy and localized plastic strain seen in the recovered cylinders exhibit strong axial symmetries which differed according to lattice orientation. Two-, three-, and four-fold symmetries are observed. We propose a simple crystallographic argument, based on the Schmid law, to understand the observed symmetries. These tests are the first to explore the role of single-crystal orientation in Taylor impact tests and they clearly demonstrate the importance of crystallography in high strain rate and temperature deformation regimes. These results provide critical data to allow dramatically improved high-rate crystal plasticity models and will spur renewed interest in the role of crystallography to deformation in dynamics regimes.

## Introduction

Most metals, when used in a technologically relevant application, are polycrystalline aggregates comprised at the micro-scale of many distinct regions of different crystallographic orientations, i.e. grains. Each of these grains consists (mostly) of a single crystallographic character that behaves, e.g. during deformation, approximately like a single crystal of material subjected to the complex constraints and boundary conditions imposed by the grains around it. Therefore, an accurate understanding of single crystal behavior is critical to predicting the bulk properties of polycrystals that arise from the collective behavior of their individual grains^1,2^. Recent high fidelity computational approaches at various length scales, i.e. molecular dynamics (MD)^3,4^ and crystal plasticity-finite element (CP-FE) models^5–7^, explicitly resolve individual grains, and use grain-scale material properties to parameterize and/or validate continuum-scale models. Although mechanical behaviors and plastic anisotropy of tantalum single crystals are well characterized in quasi-static regimes^8–10^, their dynamic behaviors at higher strain rates are less well-known.

In this report, we show that high-rate single crystal response is substantially more anisotropic, i.e. dependent on crystallographic orientation, than either quasi-static experiments on single crystals or high-rate experiments on polycrystals would suggest. This information is crucial for not only building a physics based, microstructure-aware model which does not require empirical fitting for every polycrystalline microstructure and texture, but also for validating predictions of microstructure-scale high-rate behavior.

**Figure 1:**
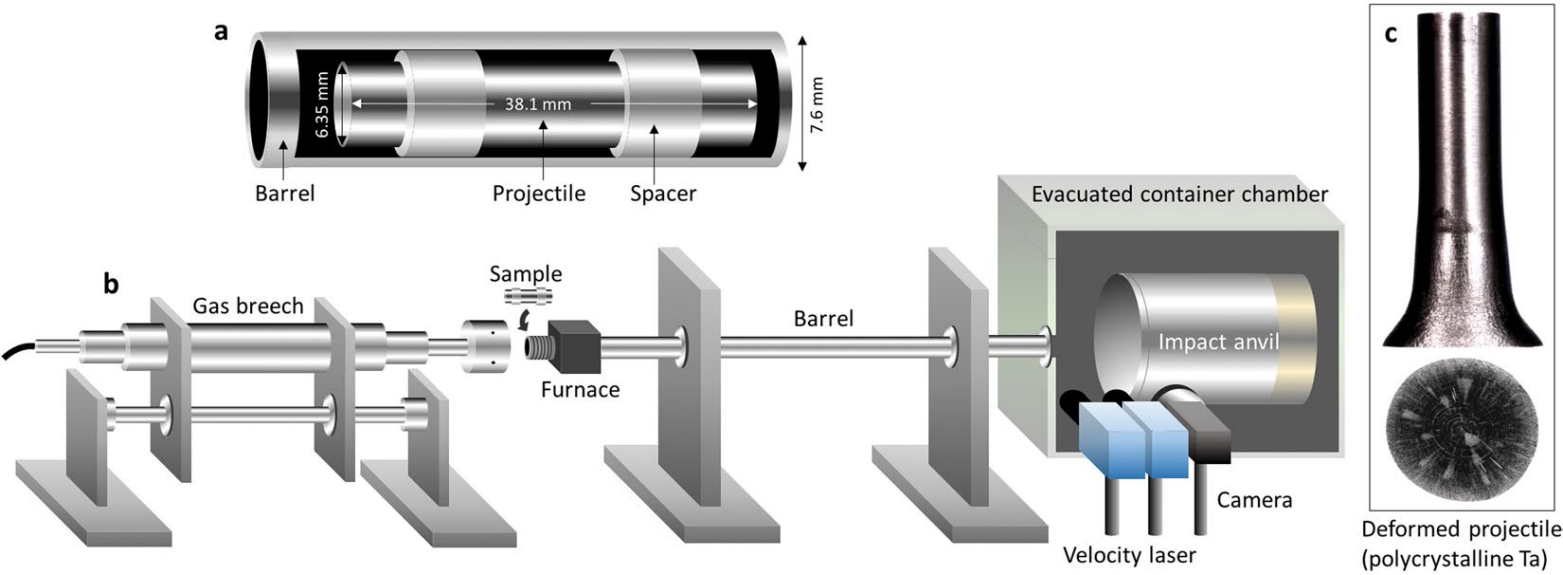
Schematic drawings of the Taylor impact experimental setup. (**a**) Single crystal tantalum projectiles with a diameter of 6.35 mm and a length of 38.1 mm are shot against the rigid surface at various velocities. (**b**) Gas gun experimental set up at Los Alamos National Laboratory. (**c**) Side and foot profiles of deformed polycrystalline tantalum projectile impacted at 146.1 m/s.

Taylor cylinder impact testing provides a simple, yet robust approach for subjecting a single specimen to a wide range of strain rates across its length^11^. In these, well known tests, a cylindrical projectile is shot into a rigid anvil at velocities in the range of 100 to 200 m/s. See the schematic description in Fig. 1, and impact movie in the online Supplementary Information. The leading end of the projectiles undergo high strain rates and temperatures while the trailing end experiences little or no deformation. The test creates large gradients of stresses and strains, which are recorded in the final shape of the deformed cylinder. Thermo-mechanical finite element simulations of polycrystalline tantalum Taylor impact experiments at 175 m/s showed that the local region with the maximum deformation can reach strain rate of 5 × 10^4^ s^−1 ^^12^ while the heat generation caused by the mechanical dissipation associated with plastic straining predict average and maximum temperatures of 405 K and 1007 K, respectively. The maximum strain rate and temperature are associated with regions of highly localized plastic deformation. Taylor impact tests provide dynamic material responses in strain-rate regimes between Split-Hopkinson pressure bar (10^2^–10^4^ s^−1^) and laser driven shock experiments (10^7^–10^9^ s^−1^). Deformation behaviors in these two regimes are generally described by dislocation plasticity theory and equation of state (EOS) theories, respectively. Taylor impact experiments are generally non-destructive, even at high-rates. Therefore, the recovered samples are especially useful in validating material models in dynamic finite element simulations^12–17^. Plastic anisotropy of the material is also captured in the cross-section of the impacted surface, i.e. the foot shape of the deformed specimen. For example, isotropic polycrystals exhibit circular foot shapes, while textured polycrystals develop oval foot shapes. Taylor impact tests have been conducted with various polycrystalline metals, i.e. tantalum^14^, copper^13^, steel alloys^13,18,19^, aluminum alloys^18^ as well as various hexagonal close-packed (hcp) metals^20–22^. However, Taylor impact tests using single crystals have not been reported previously. This work demonstrates characteristics of high rate deformation behaviors in single crystals, severe strain localization and plastic anisotropy.

## Results

Tantalum is a refractory body-centered cubic (bcc) metal that is often used in extreme environments such as nuclear, and ballistic applications. In this work, Taylor impact experiments are conducted with tantalum single crystals in [100], [110], [111] and [$\bar{1}49$] orientations aligned with the impact direction. Figure 1 shows a schematic drawing of initial sample dimensions, Taylor impact experimental setup and a representative deformed polycrystalline tantalum projectile.

**Figure 2:**
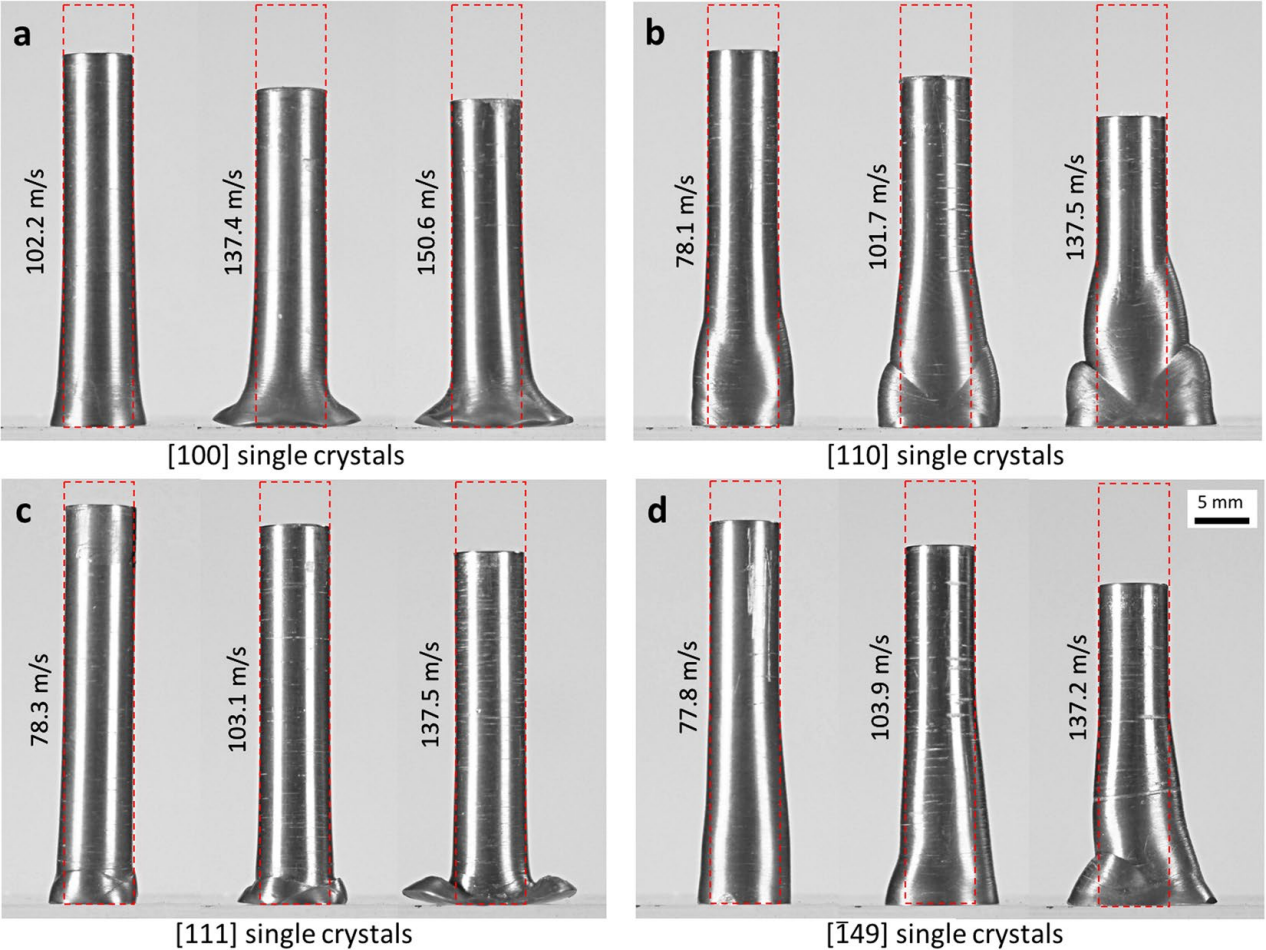
Deformed single crystal tantalum projectiles at various impact velocities. (**a**) [100] single crystals, (**b**) [110] single crystals, (**c**) [111] single crystals and (**d**) [$\bar{1}49$] single crystals. Red dashed lines represent the initial dimension of a specimen before the impact.

Figure 2 shows deformed single crystal samples after impact at various velocities. Red dashed lines represent the initial dimension of a specimen before the impact. Side profiles of deformed projectiles are significantly different from that of the polycrystalline projectile, Fig. 1. In contrast to the polycrystalline projectile that shows smooth and continuous deformation, single crystals exhibit more localized deformation, especially at higher velocities. For example, [110] single crystal at 101.7 m/s started to exhibit X-shaped shear bands and cusps - even forming ‘shoulders’ on the side of the projectile. At 137.5 m/s, subsequent deformation is strongly localized in two shoulders. In [111] single crystals, shear bands and strain localization are observed near the impact plane even at low velocity (78.3 m/s) even while the material above the shear band showed little or no deformation. [$\bar{1}49$] single crystals show an asymmetric side profile, with only one shoulder formed from localized strain at 137.2 m/s. The localization of plastic deformation and shear are more pronounced at higher impact velocities in [110], [111] and [$\bar{1}49$] crystals while they are not clearly observed in [100] single crystals. At similar impact velocities, [111] and [110] single crystals were the longest and shortest after the impact, respectively. The length of the deformed cylinder is closely related to the amount of deformation throughout the sample from the impact surface, i.e. [111] single crystals showed highly localized deformation near the impact surface while [110] single crystals exhibited relatively large deformation along the radial direction of the cylinder, up to a half the length of the cylinder. Detailed dimensions of deformed projectiles can be found in Supplementary Information, Table 1.

**Figure 3:**
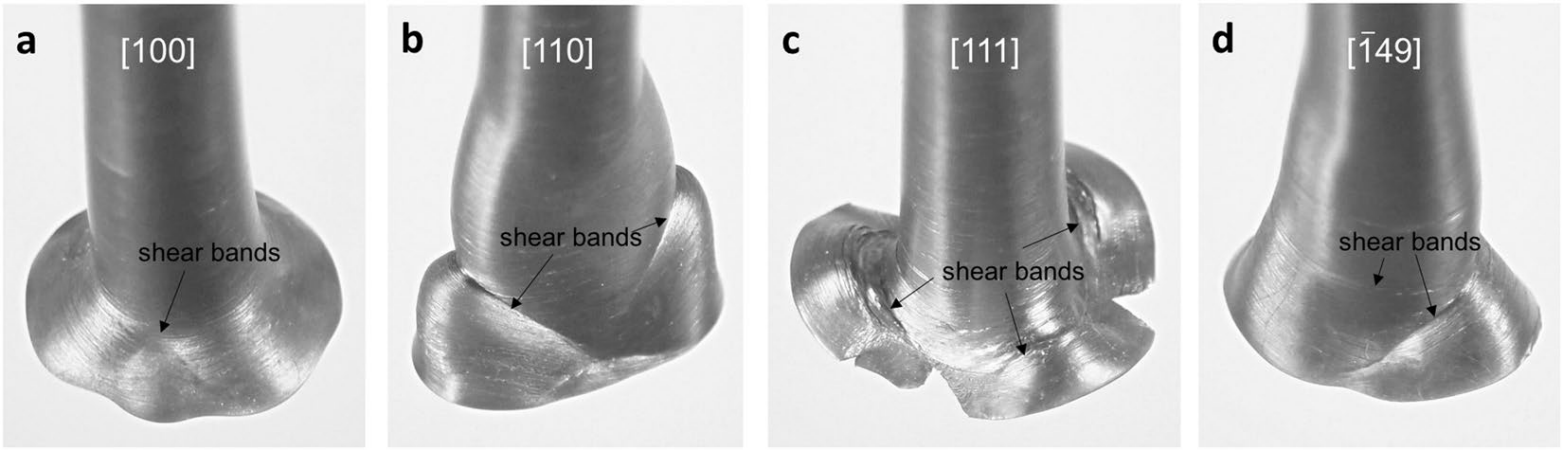
Deformed single crystal projectiles near the impact surface. (**a**) [100] single crystal at 150.6 m/s, (**b**) [110] single crystal at 137.5 m/s, (**c**) [111] single crystal at 137.5 m/s and (**d**) [$\bar{1}49$] single crystal at 137.2 m/s. Shear bands are observed near the foot of the projectile (red dashed lines).

**Figure 4:**
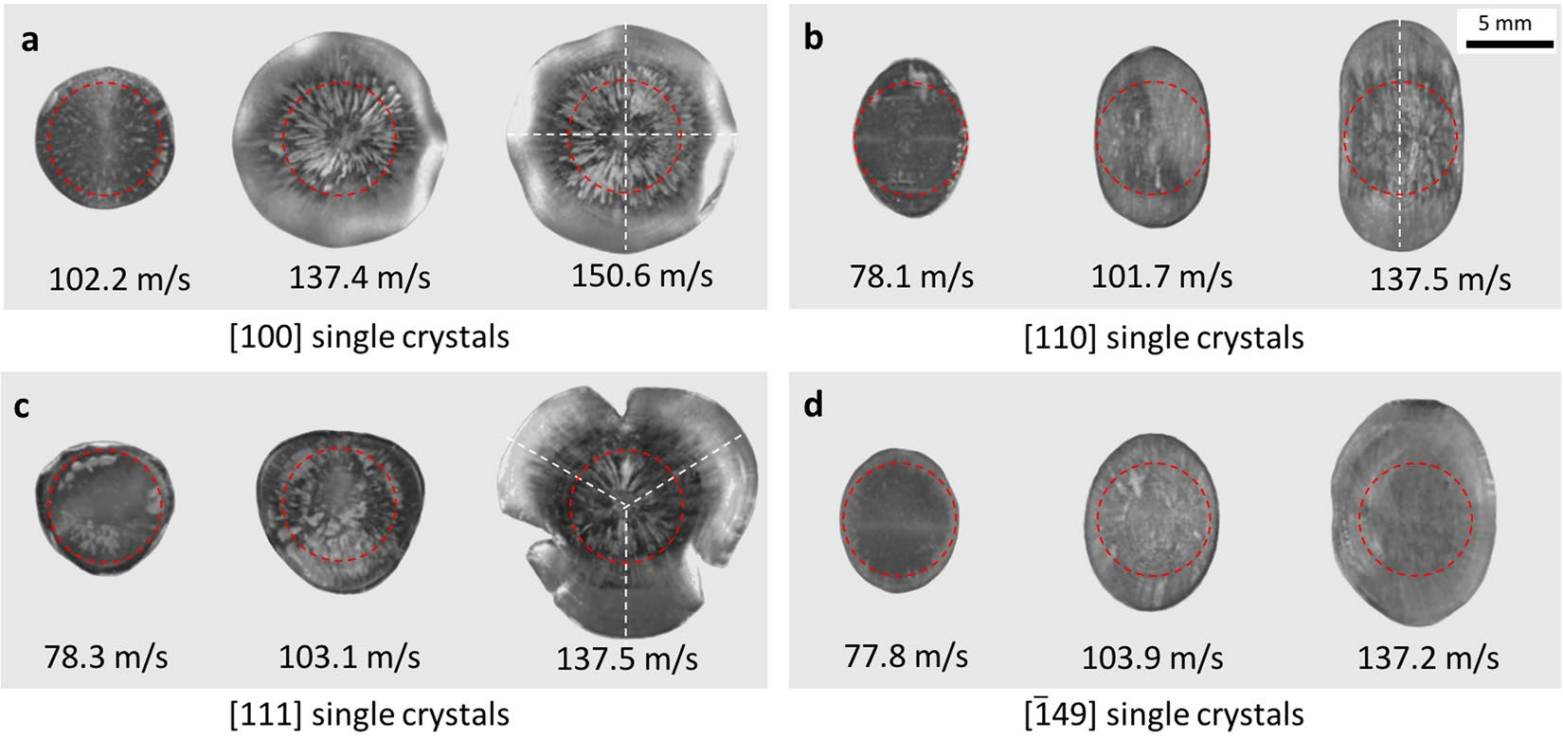
Twelve deformed foot shapes of four single crystal orientations after impact at three impact velocities each. (**a**) [100] single crystals, (**b**) [110] single crystals, (**c**) [111] single crystals and (**d**) [$\bar{1}49$] single crystals. White dashed lines represent the four, two and three-fold symmetries of a foot profile in [100], [110] and [111] single crystals after the impact. Red dashed lines represent the initial dimension of a specimen before the impact.

Figures 3 and 4 show deformed foot shapes of single crystal projectiles after the impact at various velocities. [100], [110] and [111] single crystals exhibit four, two and three-fold symmetries in their impact surfaces, respectively. Symmetries of the foot shapes are represented by white dashed lines in Fig. 2. The cross-section of [100] single crystals show eight corners, similar to octagon, but four ridges are developed above the foot of the cylinder as shown in Fig. 3. In [110] single crystals, the bottom of the projectile deformed mostly along one direction (major axis) while little deformation occurred along the minor axis. [111] single crystals show clear three-fold symmetry and relatively large deformation along the radial direction. At 137.5 m/s, the bottom surface cracked and split in three directions. Symmetries of the foot shape in [100], [110] and [111] single crystals are more noticeable at higher impact velocities. In contrast, [$\bar{1}49$] single crystals show no clear symmetry in their foot profiles. Optical images in Fig. 4 show clear radial striations in [100] and [111] single crystals compared to [110] and [$\bar{1}49$] specimens.

In order to understand the observed symmetries in foot shapes of deformed single crystals, crystallographic slip systems and their Schmid factors were calculated. The Schmid law describes the relationship between the applied stress (*σ*) and the resolved shear stress (*τ*) using slip directions and slip plane normal directions as follows:

1 $$\tau =M\sigma =\sigma \,\cos \,\varphi \,\cos \,\chi ,$$

where *M* is the Schmid factor, *ϕ* is the angle between the slip direction and the loading axis and χ is the angle between the slip plane normal and the loading axis. *M* can be obtained for each set of slip plane and slip direction. The slip system(s) with maximum *M* value will be the most favored under uniaxial loading. While close-packed cubic metals follow well-defined slip systems (i.e. face-centered cubic (fcc) on twelve 〈111〉{110} slip systems), active slip planes in bcc metals are more controversial. While it is generally accepted that slip directions are along 〈111〉 directions, many atomistic simulations and experiments on tantalum support the theory of plastic slip on {110} planes^9,23–29^ but evidence for slip on {112} or both {110} and {112} planes is also observed^30^. Other deformation mechanisms in bcc metals, such as twinning and non-Schmid effects, are expected to be insignificant at the temperatures and strain rates of Taylor impact experiments. For example, deformation twinning in impact test of tantalum has only been directly observed at temperatures below 77 K^31^ in strain rates below the shock regime, while non-Schmid behavior become insignificant at temperatures above room temperature^32–34^.

**Figure 5:**
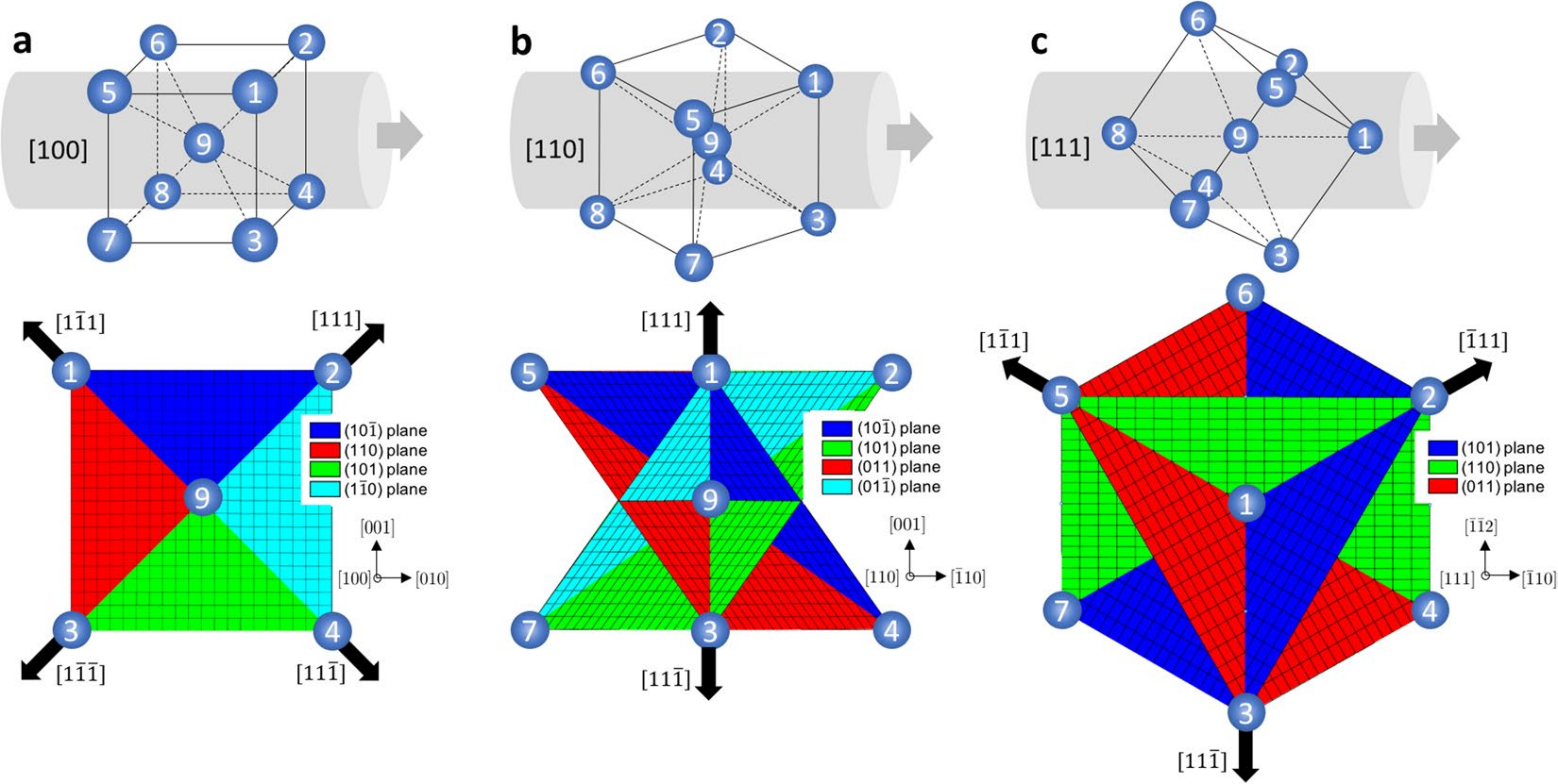
Crystal orientations of three single crystals (top) and active slip planes and directions projected to impact surface (bottom). (**a**) [100] single crystals have four active slip planes and slip directions, (**b**) [110] single crystals have four active slip planes and two slip directions, (**c**) [111] single crystals have three active slip planes and slip directions. Black arrows indicate slip directions having the maximum Schmid factors. Each of the slip directions (black arrow) is associated with two slip planes.

The maximum *M* values for [100], [110], [111] and [$\bar{1}49$] single crystals upon uniaxial loading are 0.408, 0.408, 0.272 and 0.5, respectively, for twelve 〈111〉{110} slip systems. Note that [100] and [110] single crystals have the same maximum *M* value while [111] and [$\bar{1}49$] have the minimum and the maximum values of *M* in 〈111〉{110} slip systems. Thus, [111] and [$\bar{1}49$] represent the hardest and softest single crystals upon uniaxial loading. [100], [110] and [111] single crystals have multiple equally activated slip systems, 8, 4 and 6 slip systems upon uniaxial loading. On the other hand, [$\bar{1}49$] crystal has one slip system with *M* = 0.5 and other slip systems have non-zero *M* values. Figure 5 illustrates [100], [110] and [111] single crystals in the global frame (top figures) and active slip planes and slip directions projected on the plane normal to the loading axis (foot plane). Active slip planes are obtained using Equation (1) with [100], [110] and [111] loading directions.

As shown in Fig. 5, [100], [110] and [111] single crystals have four, two and three projected slip directions that have the maximum *M* values, respectively, consistent with the foot symmetries of the projectile. Note that this also holds for {112} slip since the analysis using twelve 〈111〉{112} slip systems also exhibit four, two and three slip directions for [100], [110] and [111] single crystals upon projection of the dominant slip systems. The maximum Schmid factors obtained from {112} slip planes are 0.471, 0.471 and 0.314 for [100], [110] and [111] single crystals, higher than {110} slip systems. Thus, if {110} and {112} are equally active, four, two and three slip systems from {112} planes would dominate the deformation. Schmid factors of four single crystals upon uniaxial compression using twelve {110} and {112} slip systems are listed in Supplementary Information, Tables 2 and 3.

This simple crystallographic analysis qualitatively describes the source of the foot symmetries and the plastic anisotropy observed in single crystals. More accurate considerations of complex stress and strain states, crystal rotations, thermal and dynamic effects as well as advanced theories on plastic instability are required to better understand the deformation mechanisms of single crystals. In particular, understanding the locations of shear band formations and strain localization in single crystal is important since these local phenomena eventually lead to ductile fracture of polycrystalline materials^35,36^. Therefore, these experimental results present significant challenges, as well as future opportunities for the computational modeling community. A comprehensive computational model must incorporate thermo-mechanical coupling, temperature and strain rate dependent constitutive model as well as crystal plasticity framework to capture the plastic anisotropy and strain localizations. Most classical high temperature and strain rate dependent strength models are based on isotropic polycrystalline materials^13,37–40^ or considers collective anisotropic behaviors by utilizing continuum-scale anisotropic yield criteria^14,15,17,21^. A more recent model explicitly incorporates a crystal plasticity framework^41^.

## Conclusions

This work has shown strong strain localization and plastic anisotropy in the dynamic response of tantalum single crystals. These non-uniform plastic deformations in single crystals are much more pronounced when compared to polycrystalline tantalum. Symmetries observed in the deformed foot profile agreed well with a crystallographic analysis that suggests the plastic deformation of tantalum single crystals at high strain rates and temperature regimes is dominated by dislocation slips along close-packed 〈111〉 directions. A more complete understanding of, and predictive capability for, the dynamic deformation behavior of single crystals, including plastic localization and anisotropy, will require crystal-level simulations with accurate temperature and strain rate dependent strength models.

We plan to conduct high-resolution experimental analyses in the near future which will allow subsequent analysis comparing different state variables from computational models, i.e. local strain fields and microstructural evolution.

## Methods

Three single crystal specimens were made per orientations (a total of 12 specimens) from Princeton Scientific Corporation and machined to have a diameter of 6.35 mm and a length of 38.1 mm. Two Teflon spacers were attached to single crystal specimens to fit in a 30-caliber 0.3 inch (7.62 mm) smooth-bore launch tube. Taylor impact tests were conducted at the impact facility in Los Alamos National Laboratory. Single crystal projectiles were shot at various velocities, ranging from 78 to 151 m/s against a pneumatically-positioned AF1410 high-strength steel anvil ground to a mirror surface finish. All experiments were performed at room temperature and vacuum conditions, typically at 10 torr. The projectile velocities were measured using four laser beams located at various locations along the traveling path. The last laser beam was placed about 1 mm before the impact. High-speed photography was added to capture 128 frames of images to reveal the deformation process in real time.

## Electronic supplementary material

Tantalum single crystal impact experiments

Supplementary information
