# Supplementary material for: Anisotropy and Strain Localization in Dynamic Impact Experiments of Tantalum Single Crystals
Source: Sci Rep. 2018 Apr 3;8:5540. doi: 10.1038/s41598-018-23879-1 (PMC5883051; doi:10.1038/s41598-018-23879-1)
Supplement: Supplementary file 2 — Supplementary information [file 41598_2018_23879_MOESM2_ESM.docx]

**Anisotropy and Strain Localization in Dynamic Impact Experiments of Tantalum Single Crystals**

**Hojun Lim, Jay D. Carroll, Corbett C. Battaile, Shuh Rong Chen, Alexander P. Moore and J. Matthew D. Lane**

**Supplementary information**

**Table 1: Dimensions of deformed [100], [110], [111] and**$\left[ \bar{\boldsymbol{1}}\boldsymbol{49} \right]$ **single crystal projectiles at various impact velocities.**

| Specimen | Velocity (m/s) | Length (mm) | Foot diameter (mm)  major/minor axis |
| --- | --- | --- | --- |
| [100] | 102.2 | 33.4 | 8.6/8.0 |
|  | 137.4 | 30.3 | 13.5/12.6 |
|  | 150.6 | 30.1 | 14.3/13.7 |
| [110] | 78.1 | 34.2 | 9.4/6.6 |
|  | 101.7 | 31.8 | 10.9/6.7 |
|  | 137.5 | 28.5 | 14.1/7.5 |
| [111] | 78.3 | 37.0 | 8.5/8.3 |
|  | 103.1 | 34.2 | 10.2/9.8 |
|  | 137.5 | 31.8 | 15.8/15.2 |
| $\left[ \bar{1}49 \right]$ | 77.8 | 34.6 | 8.4/7.0 |
|  | 103.9 | 33.1 | 10.7/8.2 |
|  | 137.2 | 29.3 | 13.9/9.5 |

**Table 2: The twelve {110} slip systems and Schmid factors (*M*) for [100], [100], [111] and** $\left[ \bar{\boldsymbol{1}}\boldsymbol{49} \right]$ **orientations upon uniaxial loading.**

| α | Slip system | *M*_[100]_ | *M*_[110]_ | *M*_[111]_ | *M*_[149]_ |
| --- | --- | --- | --- | --- | --- |
| 1 | $\left( 01\bar{1} \right)\left[ 111 \right]$ | 0 | 0.4082 | 0 | 0.2500 |
| 2 | $\left( \bar{1}01 \right)\left[ 111 \right]$ | 0.4082 | 0.4082 | 0 | 0.5000 |
| 3 | $\left( 1\bar{1}0 \right)\left[ 111 \right]$ | 0.4082 | 0 | 0 | 0.2500 |
| 4 | $\left( \bar{1}0\bar{1} \right)\left[ \bar{1}11 \right]$ | 0.4082 | 0 | 0.2722 | 0.4667 |
| 5 | $\left( 0\bar{1}1 \right)\left[ \bar{1}11 \right]$ | 0 | 0 | 0 | 0.2916 |
| 6 | $\left( 110 \right)\left[ \bar{1}11 \right]$ | 0.4082 | 0 | 0.2722 | 0.1750 |
| 7 | $\left( 0\bar{1}\bar{1} \right)\left[ \bar{1}\bar{1}1 \right]$ | 0 | 0.4082 | 0.2722 | 0.3250 |
| 8 | $\left( 101 \right)\left[ \bar{1}\bar{1}1 \right]$ | 0.4082 | 0.4082 | 0.2722 | 0.2000 |
| 9 | $\left( \bar{1}10 \right)\left[ \bar{1}\bar{1}1 \right]$ | 0.4082 | 0 | 0 | 0.1250 |
| 10 | $\left( 10\bar{1} \right)\left[ 1\bar{1}1 \right]$ | 0.4082 | 0 | 0 | 0.1667 |
| 11 | $\left( 011 \right)\left[ 1\bar{1}1 \right]$ | 0 | 0 | 0.2722 | 0.2167 |
| 12 | $\left( \bar{1}\bar{1}0 \right)\left[ 1\bar{1}1 \right]$ | 0.4082 | 0 | 0.2722 | 0.0500 |

**Table 3: The twelve {112} slip systems and Schmid factors (*M*) for [100], [100], [111] and** $\left[ \bar{\boldsymbol{1}}\boldsymbol{49} \right]$ **orientations upon uniaxial loading.**

| α | Slip system | *M*_[100]_ | *M*_[110]_ | *M*_[111]_ | *M*_[149]_ |
| --- | --- | --- | --- | --- | --- |
| 1 | $\left( 11\bar{2} \right)\left[ 111 \right]$ | 0.2357 | 0.4714 | 0 | 0.4329 |
| 2 | $\left( \bar{2}11 \right)\left[ 111 \right]$ | 0.2357 | 0.2357 | 0 | 0 |
| 3 | $\left( 1\bar{2}1 \right)\left[ 111 \right]$ | 0.4741 | 0.2357 | 0 | 0.4329 |
| 4 | $\left( \bar{1}1\bar{2} \right)\left[ \bar{1}11 \right]$ | 0.4741 | 0 | 0.3143 | 0.3704 |
| 5 | $\left( \bar{1}\bar{2}1 \right)\left[ \bar{1}11 \right]$ | 0.2357 | 0 | 0.1571 | 0.0673 |
| 6 | $\left( 211 \right)\left[ \bar{1}11 \right]$ | 0.2357 | 0 | 0.1571 | 0.4377 |
| 7 | $\left( \bar{1}\bar{1}\bar{2} \right)\left[ \bar{1}\bar{1}1 \right]$ | 0.4714 | 0.2357 | 0.1571 | 0.0433 |
| 8 | $\left( 2\bar{1}1 \right)\left[ \bar{1}\bar{1}1 \right]$ | 0.2357 | 0.2357 | 0.1571 | 0.2598 |
| 9 | $\left( \bar{1}21 \right)\left[ \bar{1}\bar{1}1 \right]$ | 0.2357 | 0.4714 | 0.3143 | 0.3030 |
| 10 | $\left( 1\bar{1}\bar{2} \right)\left[ 1\bar{1}1 \right]$ | 0.4714 | 0 | 0.1571 | 0.0673 |
| 11 | $\left( 121 \right)\left[ 1\bar{1}1 \right]$ | 0.2357 | 0 | 0.3143 | 0.1539 |
| 12 | $\left( \bar{2}\bar{1}1 \right)\left[ 1\bar{1}1 \right]$ | 0.2357 | 0 | 0.1571 | 0.2213 |
